# Effects of Interleukin 2 Receptor Blockers on Patient and Graft Survival in Renal-Transplanted Children

**DOI:** 10.5812/numonthly.18641

**Published:** 2014-07-05

**Authors:** Mostafa Sharifian, Banafsheh Arad, Naser Simfroosh, Abbas Basiri, Hassan Otukesh, Nasrin Esfandiar

**Affiliations:** 1 Pediatric Infections Research Center, Shahid Beheshti University of Medical Sciences, Tehran, IR Iran; 2 Iran University of Medical Sciences, Tehran, IR Iran

**Keywords:** Receptors, Interleukin-2, transplantation, Child

## Abstract

**Background:**

Monoclonal antibodies block interleukin-2 receptors on alloantigen-reactive T-Lymphocytes and induce selective immunosuppression. It is postulated that induction therapy with these agents in pediatric transplantation may decrease acute rejection and improve graft survival with no significant side effect or increase in the incidence of viral infections.

**Objectives:**

The aim of this study was to examine the effects of interleukin 2 receptor blockers on patient and graft survival in renal-transplanted children.

**Patients and Methods:**

One hundred and eighty six children aged 7-13 years who received renal transplantation in university-affiliated hospital between 2003 and 2012 were enrolled in the study. All patients received prednisolone, cyclosporine and mycophenolate mofetil or azathioprine as basic immunosuppressive therapy. Patients were divided into two groups according to receiving induction therapy with IL2-receptor blockers. We investigated for acute rejection episodes, Cytomegalovirus (CMV) and BK virus infection and one and three year’s survival of the patients and the grafts

**Results:**

From 186 renal-transplanted children included in this study, 36 patients were in treated group (group 1) and 150 patients in control group (group 2). The mean age of the patients was 10.4 ± 2 years and 55.6% were males. In first six months of transplantation, eight patients in group one had one episode of acute rejection and no one had two episodes. Early acute rejection rate was 8.36 (22%). In the control group, 37 patients had one episode and three patients had two episodes of acute rejection (rejection rate 28.6%). Therefore, early acute rejection rates were lower in group one. Late acute rejection rates did not show any difference in group 1 and group 2 (27.7% vs. 27.3% respectively). There was lower prevalence of steroid-resistance rejection in group 1 patients (5.5%) compared with 6.6% in group 2, but it did not reach statistical significance. None of the patients in IL2-R blocker group died at one year follow-up (patient survival 100%). However, in control group, four (2.6%) patients died toward the end of first year (patient survival 97.4%). When patients in group 1 and group 2 were age and sex matched with equal number the difference was significant (P < 0.05).

**Conclusions:**

Induction therapy with IL2-R blockers reduced the rate of early acute rejection, but had no effect on late acute rejections. Patient and graft survival were better in treated group, but did not reach statistical significance. A longer period of follow-up may be required to discern a clear advantage for induction therapy with these agents.

## 1. Background

Renal transplantation is the optimal therapy for children with end-stage renal failure (1). Acute rejection is one of the strongest predictor of long-term graft survival (2). According to North American Pediatric Renal Trials and Collaborative Studies (NAPRTCS) reports in 2006, acute rejection was cause of 13% of graft failure (3). IL2-Receptor (IL2-R) blockers are selective induction agents e.g. Basiliximab is a recombinant and Daclizumab a humanized recombinant antibodies against IL2-R α-chain, also known as CD_25_ antigen on the surface of activated T lymphocytes. These agents inhibit proliferation of T cells and therefore cytokines release (4). Basiliximab saturates CD_25_ subunits for five to eight weeks after transplantation (5). Daclizumab provide three months extra immunosuppression in patient receiving steroids as basic immunosuppressive therapy (4). These agents have no important side effects and are well-tolerated in children (6, 7).

In a randomized controlled trials of IL2-R blockers induction, Basiliximab and Daclizumab decrease acute rejection compared to placebo by 28-46% within the first year after transplant (8-12), but were not associated with improving one year graft survival (7-14). With reference to 2006 NAPRTCS data, 28.6% of patients received Basiliximab and 20.6% received Daclizumab for induction, but it reduced to 21.5% for Basiliximab and 16.3% for Daclizumab in 2010 (15). Recently, IL2-R blockers have been used to reduce or eliminate steroids in standard immunosuppression protocols (16, 17).

In early period after renal transplantation, we face with many medical complications including Acute Tubular Necrosis (ATN), vascular thrombosis and acute rejection. If IL2-R blockers reduce acute rejection at this time, it would be helpful for saving the graft.

## 2. Objectives

The aim of this study was to evaluate the effect of IL2-R blockers on acute rejection episodes and one-year patient and graft survival in kidney transplant children.

## 3. Patients and Methods

This longitudinal observational study analyzed data from 214 children who received first renal transplant between years 2003 and 2012 in Labbafinejhad Hospital, Tehran; affiliated to Shahid Beheshti University of Medical Sciences. All patients received Prednisolone, cyclosporine and azathioprine/mycophenolate mofetil as basic immunosuppressive therapy. Two groups were defined based on receiving or not receiving IL2-R blockers. Forty-two patients received IL2-R blockers (nine patients, daclizumab and 33 patients, basiliximab). Basiliximab was given on day 0 and day 4 of the transplant, 10 mg/dose in children under 35 kg and 20 mg/dose in heavier patients. Daclizumab was given on day zero then every two weeks for a total of five doses at 1 mg/kg/dose. Patients were followed at least monthly in transplant clinic of the hospital. Twenty-eight patients were excluded from the study because of lost follow up in the first year after transplant (six patients in treated group and 22 patients in control group). Therefore, 186 patients included in the study (36 in treated group and 150 in control group). In treated group, seven patients received daclizumab and 29 patients received basiliximab. The primary end-point was death and graft loss and secondary outcomes were rejection and incidence of viral infections. Acute rejection was defined as any significant increment in serum creatinine (more than 10% from baseline), low-grade fever or new onset of hypertension without evidence of infection, which was treated with methyl prednisolone pulses for 3-5 days. No response to this therapy was considered as steroid resistant rejection and was treated with anti-thymocyte globulin (ATG). The Schwartz formula was used for estimation of glomerular filtration rate (GFR). Cytokmegalovirus (CMV) infection detected with plasma PCR and Ag pp65, BK-virus with plasma and urine PCR and varicella infection with clinical manifestations.

### 3.1. Statistical Analyses

Variables analyzed were induction therapy, basic immunosuppressive therapy, type of donor, donor and recipient age, donor and recipient gender, recipient blood group, pre-transplant dialysis, pre-transplant transfusion, cause of death and graft failure, creatinine before and after transplantation and GFR in the last visit. Chi square analysis was used to compare the incidence rate of acute rejection between treatment regimen groups. For comparison of mean values, the Student's t-test was used. Results were expressed as mean ± standard deviation and differences were considered significant when 2-sided P Value was less than 0.05. Statistical analyses were performed using SPSS software, version 16.

## 4. Results

**Figure 1.**
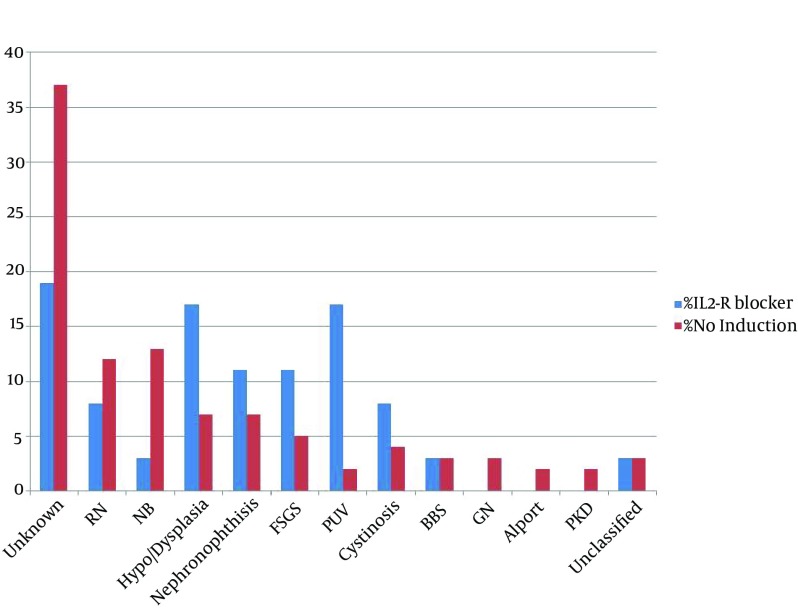
Primary Renal Disease of 186 Patients Underwent Renal Transplantation IL2-R blocker (n = 36) vs. no induction (n = 150) groups. P: NS. RN = Reflux Nephropathy, NB = Neurogenic Bladder, FSGS = Focal Segmental Glomerulosclerosis, PUV = Posterior Urethral Valve, BBS = Bardet-Biedl Syndrome, GN = Glomerulonephritis and PKD = Polycystic Kidney Disease. NS = Not significant.

Out of or of 186 children, 55.6% were male. The mean age of the patients was 10.4 ± 2 years. The youngest patient was a three-years-old boy. IL2-R blocker receiving patients included 36 patients (Group 1), while 150 patients did not receive this induction therapy (Group 2). Table 1 depicted the cause of end-stage renal failure of the two study groups. In unclassified group, three patients had obstructive uropathy, one patient had atypical Hemolitic-Uremic Syndrome (HUS), one patient had congenital nephrotic syndrome and one had primary hyperoxaluria, whom was diagnosed after transplantation. Primary renal disease of patients is shown in Figure 1 and demographic characteristics of the two study groups are shown in Table 1. Groups did not have any significant difference in demographic characteristics; however, the mean age of treated group was lower than control group.

**Table 1.** Demographic and Clinical Characteristics of Patients Underwent Renal Transplantation: IL2-R Blockers vs. no Induction (P: NS)^a^

Characteristics	IL2-R Blocker (n = 36)	No Induction (n = 150)
**Age in years, mean ± SD**	9.3 ± 2.7	10.7 ± 2.7
**Male recipients, No. (%)**	25 (69.4)	77 (51.3)
**Blood group, No. (%)**		
O^+^	9 (25)	50 (33.3)
A^+^	10 (27.7)	40 (26.6)
B^+^	8 (22.2)	38 (25.3)
AB^+^	5 (13.8)	14 (9.3)
A^-^	2 (5.5)	3 (2)
O^-^	1 (2.7)	3 (2)
B^-^	1 (2.7)	2 (1.3)
**Donor age in years, mean ± SD**	27.3 ± 4.8	27.4 ± 5.6
**Pre-Tp Dialysis, No. (%)**	16 (44.4)	71 (47.3)
**Pre-emptive HD**	10 (27.7)	68 (45.3)
**CAPD**	10 (27.7)	11 (7.3)
**Pre-Tp Transfusion, No. (%)**	3 (8.3)	29 (19.3)
**Creatinine before Tp, mean ± SD**	8 ± 2.8	7.4 ± 2.7

^a^ Abbreviations: MMF, mycophenolate mofetil; CsA, cyclosporine A; Pred, prednisolone; Tp, transplant; PRE, preemptive; HD, hemodialysis; CAPD, continuous ambulatory peritoneal dialysis; HTN, hypertension.

### 4.1. Rejection

In the first six months of transplantation, eight patients in group one had one episode of acute rejection and no one had two episodes; thus, early acute rejection rate was 8 (22%). In group two, 37 patients had one episode and three patients had two episode of acute rejection; thus, early acute rejection rate was 43 (28.6%), therefore early acute rejection rates were lower in group one compared with group two. Late acute rejection rates did not show any difference in both groups (27.7% vs. 27.3%). There was lower prevalence of steroid-resistant rejection in group one (5.5%) compared with patients in group two (6.6%), but it did not reach statistical significance. Table 2 depicts the comparison between two groups in overall clinical outcome.

**Table 2.** Clinical Outcome of Patients Underwent Renal Transplantation: IL2-R Blocker vs. no Induction^a,b^

Characteristics	IL2-R Blocker (n = 36)	No Induction (n = 150)	P Value
**Early acute rejection**	8 (22.2)	43 (28.6)	NS
**Late acute rejection**	10 (27.7)	41 (27.3)	NS
**Steroid-resistance rejection**	2 (5.5)	10 (6.6)	1
**Creatinine after Tp**	1.1 ± 1.2	1.1 ± 1.1	0.93
**GFR at last visit**	65.3 ± 29.6	59.7 ± 31.7	0.32
**Graft loss**	3 (8.3)	14 (9.3)	0.65
**Patient death**	0	4 (2.6)	0.39
**CMV infection**			
At first 6 months	4 (11.1)	12 (8)	1
After 6 months	2 (5.5)	7 (4.6)	
**BK-virus infection**			
At first 6 months	1 (2.7)	2 (1.3)	0.57
After 6 months	2 (5.5)	4 (2.6)	

^a^ Abbreviation: Tp, Transplantation.

^b^ Data are presented as Mean ± SD or No. (%).

### 4.2. Graft Survival

Three patients in group one had graft failure toward the end of first year, (one of them due to non-compliance, one with vascular thrombosis and one due to infection). In group two, 14 patients had graft failure, (seven patients had rejection, two patients with recurrence of the primary disease, two patients had thrombosis, two patients due to infection and one because of HUS post transplantation).

Induction with IL2-R blockers did not significantly improve graft one year survival, although there was a trend toward positive benefit without statistical significant (8.3% vs. 9.3% in group one and two, respectively, P = 0.65, Table 2).

### 4.3. Patient Survival

None of the patients in IL2-R blocker group expired in our one-year follow-up. However, in control group, four (2.6%) patients died toward the end of first year. Patient survival at one year was not significantly difference in the two groups of patients (P = 0.39, Table 2), However when patients in group one and two were age and sex matched with equal number, the mortality difference was significant (P < 0.05).

Acute tubular necrosis, due to acute rejection and complications of Acute Renal Failure (ARF) in two patients, cardiovascular complications in one and sepsis in another, were cause of death of these patients.

### 4.4. Renal Function

At one year, there was no significant difference in renal function between two groups of patients.

### 4.5. Viral Infections

Among patients in IL2-R blocker group, 16.6% developed CMV infection, 8.2% developed BK-virus infection and no patient developed smallpox, in control group 12.6% developed CMV infection, 3.9% developed BK-virus and 2% Varicella toward the end of the first year. The viral infection at one year was not significantly different in two groups of patients. None of the study patients in either group developed malignancy.

## 5. Discussion

In this study, we report one-year follow up analysis of the effect of IL2-R blockers on acute rejection and graft and patient survival after renal transplantation. IL2-R blockers reduced the rate of early acute rejection episodes compared with no induction (28.6% for IL2-R blockers vs. 22.2% for no induction), consistent with the findings of prior randomized trials (8-12, 18-20), meta-analysis (13, 21) and retrospective data base analyses (2, 22).

IL2-R blockers reduced the severity of acute rejection compared to no induction, but the difference was not significant (5.5% vs. 6.6%, P = 1). There was not any difference in late acute rejection rate between the two groups; it may be because of larger number of haplotype-matched patients in treated group. Also in control group, 7 (4.6%) had graft loss because of rejection, but was not the cause of graft loss in treated patients.

Vincenti et al. reported one-year graft survival in their patients that was 95% in Basiliximab group and 90% in placebo group; it is comparable to our study which was 91.7% and 90.7% respectively (11). Atlani et al. also did not find any significant difference in patient survival at one year between their two study groups (2). Similar outcome have been reported by Webster et al. (13), Sheashaa et al. (10) and Patlolla et al. (23). In 2010 NAPRTCS annual report, one-year patients' survival rate for hosts receiving kidney from living donor was 98.4 ± 0.2 percent and one-year graft survival in this population was 95%. In our study it was 97.8% and 91.3%, respectively, in 186 patients (15).

IL2-R blocker induction reduces the risk of acute rejection in first six months after transplantation in renal-transplanted children. These drugs improve one and three year's patient survival. However, the drug did not improve one-year graft survival. A longer period of follow up may be required to discern a clear advantage for induction therapy with these agents.
